# Genetically encoded fluorescent indicators for imaging intracellular potassium ion concentration

**DOI:** 10.1038/s42003-018-0269-2

**Published:** 2019-01-14

**Authors:** Yi Shen, Sheng-Yi Wu, Vladimir Rancic, Abhi Aggarwal, Yong Qian, Shin-Ichiro Miyashita, Klaus Ballanyi, Robert E. Campbell, Min Dong

**Affiliations:** 1 Department of Urology, Boston Children’s Hospital, Department of Microbiology and Immunobiology, Department of Surgery, Harvard Medical School, 300 Longwood Avenue, Boston, MA 02115 USA; 2 Department of Chemistry, University of Alberta, Edmonton, AB T6G 2G2 Canada; 3 Department of Physiology, University of Alberta, Edmonton, AB T6G 2H7 Canada; 4 Department of Chemistry, The University of Tokyo, Tokyo, 113-0033 Japan

## Abstract

Potassium ion (K^+^) homeostasis and dynamics play critical roles in biological activities. Here we describe three genetically encoded K^+^ indicators. KIRIN1 (potassium (K) ion ratiometric indicator) and KIRIN1-GR are Förster resonance energy transfer (FRET)-based indicators with a bacterial K^+^ binding protein (Kbp) inserting between the fluorescent protein FRET pairs mCerulean3/cp173Venus and Clover/mRuby2, respectively. GINKO1 (green indicator of K^+^ for optical imaging) is a single fluorescent protein-based K^+^ indicator constructed by insertion of Kbp into enhanced green fluorescent protein (EGFP). These indicators are suitable for detecting K^+^ at physiologically relevant concentrations in vitro and in cells. KIRIN1 enabled imaging of cytosolic K^+^ depletion in live cells and K^+^ efflux and reuptake in cultured neurons. GINKO1, in conjunction with red fluorescent Ca^2+^ indicator, enable dual-color imaging of K^+^ and Ca^2+^ dynamics in neurons and glial cells. These results demonstrate that KIRIN1 and GINKO1 are useful tools for imaging intracellular K^+^ dynamics.

## Abstract (web summary)

Yi Shen et al. report two classes of genetically encoded fluorescent potassium indicators, based on FRET (KIRIN1 and KIRIN1-GR) or single-wavelength fluorescence change (GINKO1). These K^+^ indicators enable imaging of intracellular K^+^ dynamics in live cells with a multiplexing potential.

## Introduction

Intracellular and extracellular potassium ion (K^+^) concentration affects all aspects of cellular homeostasis^1^. Normal levels of K^+^ concentration (~150 mM for intracellular K^+^; ~5 mM for extracellular K^+^) are vital for the proper functioning of neuronal^2,3^, cardiovascular^4^, and immune systems^5–7^. Abnormal K^+^ concentration levels are often associated with disease conditions^8,9^. Measuring K^+^ concentration has predominantly relied on K^+^-specific glass capillary electrodes^10^. Although sensitive and accurate, such electrode-based measurements are invasive, time consuming, and low throughput. Electrode-based measurements also provide little to no spatiotemporal information on K^+^ dynamics in biological samples. Alternatively, synthetic small molecule-based K^+^-sensitive fluorescent dyes have been developed, but these dyes usually have poor selectivity and bind to Na^+^ with similar affinity^11,12^. K^+^-sensitive dyes with improved selectivity have been recently reported^13,14^, but the use of synthetic dyes still involves cumbersome loading and washing steps. In addition, it is generally impractical to target synthetic dyes to specific cells within a tissue.

Much as genetically encoded calcium ion (Ca^2+^) indicators have revolutionized the study of cell signaling and Ca^2+^ biology in vivo, so might genetically encoded K^+^ indicators revolutionize the study of K^+^ homeostasis and dynamics in live cells and in vivo. Genetically encoded K^+^ indicators would allow accurate measurement of K^+^ concentration in specific cell types or cellular organelles with high spatial and temporal resolution. The key to designing such indicators is to identify or develop a suitable sensing domain with a high degree of specificity toward K^+^ as well as sufficient levels of conformational change upon binding to K^+^. Recently, an *Escherichia* *coli* K^+^ binding protein (Kbp) was identified and structurally characterized^15^. Kbp is a small (149 residues, 16 kDa) cytoplasmic protein that binds K^+^ with high specificity. It contains two domains: BON (bacterial OsmY and nodulation)^16^ at the N terminus and LysM (lysin motif)^17^ at the C terminus. Small-angle X-ray scattering structural analysis of Kbp revealed that the protein exhibits a global conformational change upon K^+^-dependent association of the BON and LysM domains^15^. Such a conformational change is an important prerequisite for developing an effective genetically encoded K^+^ indicator.

Genetically encoded indicators have been widely used for studying various biochemical activities in live cells^18,19^. Among these indicators, intramolecular Förster resonance energy transfer (FRET)-based indicators are particularly useful for detecting binding-induced protein conformational changes^20^. The design principle of such indicators is straightforward and well established: a sensing domain is attached to two fluorescent proteins as a single polypeptide chain. Upon analyte binding, the conformational change of the sensing domain affects the FRET efficiency between the attached fluorophores, thus altering the ratiometric fluorescence emission^21^. FRET ratio is independent of protein expression levels, and therefore it can be utilized for quantitative imaging in live cells. A drawback of FRET-based indicators is that they each span a large portion of the visible spectrum due to the employment of two fluorescent proteins, limiting their applications in multiplexed imaging experiments. Single fluorescent protein-based indicators typically utilize the conformational change of the sensing domain to allosterically alter the fluorescent protein chromophore environment, resulting in an intensiometric change in fluorescence^22^. Due to their narrower spectral profiles, single fluorescent protein-based indicators are more suitable for multiparameter imaging^23^.

The development of fluorescent protein-based genetically encoded indicators for Ca^2+^, Zn^2+^, Mg^2+^, adenosine triphosphate (ATP), neurotransmitters, membrane voltage, and various enzyme activities has led to numerous new biological insights^24–32^. However, analogous indicators for monovalent metal cations have been absent from this toolkit until the recent report of FRET-based potassium (K^+^) indicators, known as GEPIIs (genetically encoded potassium ion indicators), based on Kbp and the mseCFP/cpVenus FRET pair^32,33^. GEPIIs were successfully used to quantify K^+^ in biological samples in vitro and to visualize K^+^ concentrations in cells^33^.

Here we report an independent and parallel effort to engineer and characterize genetically encoded K^+^ indicators based on Kbp. We describe both a cyan-yellow potassium (K) ion ratiometric indicator (KIRIN1) and green-red (KIRIN1-GR) FRET-based K^+^ indicators, as well as a single fluorescent protein-based green indicator of K^+^ for optical imaging (GINKO1). Considering their independent origins, KIRIN1 and GEPII are remarkably similar in terms of design and performance, although different donor fluorescent proteins were utilized in KIRIN1 and the GEPIIs. KIRIN1-GR is a spectrally distinct FRET-based indicator, while GINKO1 is single fluorescent protein-based K^+^ indicator. These indicators enable imaging of intracellular K^+^ concentrations in live mammalian cell lines and dissociated neurons as proof-of-principle demonstrations.

## Results

### Development of FRET-based K^+^ indicators

**Fig. 1:**
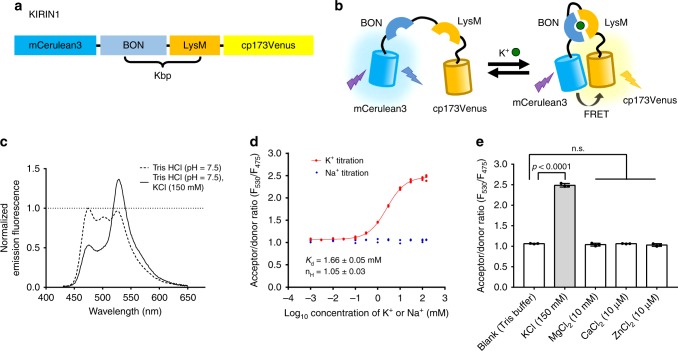
Design and in vitro characterization of KIRIN1. **a** Design and construction of the genetically encoded K^+^ indicator KIRIN1. mCerulean3 (residues 1–228) at the N terminus is linked with Kbp (residues 2–149), followed by cp173Venus (full length) at the C terminus. **b** Schematic representation of the molecular sensing mechanism of KIRIN1. Binding of K^+^ induces a structural change in the Kbp protein, increasing the FRET efficiency between mCerulean3 and cp173Venus in KIRIN1. **c** KIRIN1 emission fluorescence spectrum (excitation 410 nm, emission 430 nm to 650 nm) in Tris HCl buffer with (solid) and without (dashed) K^+^. **d** FRET acceptor-to-donor fluorescence ratio (*F*_530_/*F*_475_) of purified KIRIN1 protein at various concentrations (1 µM to 150 mM) of K^+^ (red) and Na^+^ (blue) solutions, data are expressed as individual data points. **e** FRET acceptor-to-donor fluorescence ratio of purified KIRIN1 protein upon addition of various physiologically relevant ions including Mg^2+^ (10 mM), Ca^2+^ (10 µM), and Zn^2+^ (10 µM), data are expressed as mean ± SD

We designed genetically encoded K^+^ indicators based on the fusion of a fluorescent protein donor–acceptor FRET pair to the termini of Kbp (Fig. 1a). Apo-Kbp adopts an elongated conformation with N/C termini relatively far away from each other; the K^+^-bound Kbp showed a globular conformation with its N/C termini close to each other^15^. Upon K^+^ binding, the conformational change of Kbp brings the fused fluorescent protein donor and acceptor closer together, thus increasing the FRET efficiency and resulting in a ratiometric fluorescence change (donor emission fluorescence decrease and acceptor emission fluorescence increase) (Fig. 1b). We constructed a fusion protein consisting of the cyan fluorescent protein (CFP) mCerulean3 as the FRET donor^34^ and a variant of yellow fluorescent protein (YFP), cp173Venus^35^, as the FRET acceptor. The donor, mCerulean3, is the brightest CFP currently available^36^ with a high quantum yield at 0.80. The acceptor, cp173Venus, is a bright FRET acceptor that has been optimized for high FRET efficiency with a CFP-based donor^35^. mCerulean3 is linked to the N terminus of Kbp (residues 2–149) and cp173Venus is attached to the C terminus (Fig. 1a, b). The resulting construct was designated KIRIN1.

To investigate the potential utility of KIRIN1 as a K^+^ indicator, the protein was purified and characterized in vitro. To determine the K^+^-induced FRET efficiency change, we first measured the emission spectrum (430 nm to 650 nm) of KIRIN1 protein in Tris buffer (Fig. 1c). Upon adding 150 mM K^+^, the mCerulean3 emission (~475 nm) decreased by ~50% and cp173Venus (~530 nm) emission increased by ~30%, indicating an increase in FRET efficiency. The maximum FRET acceptor and donor fluorescence emission intensity ratio (*R* = *F*_530_/*F*_475_) change (Δ*R*/*R*_min_) was 130%, which is a relatively large change compared to other first-generation CFP-YFP-based FRET sensors. However, it is not as large as changes for highly optimized FRET-based indicators^35,37^.

To determine the K^+^ affinity of KIRIN1, we measured the mCerulean3 and cp173Venus fluorescence emission ratio (*F*_530_/*F*_475_) in the presence of K^+^ concentrations ranging from 1 µM to 150 mM (Fig. 1d). The indicator showed FRET ratio changes over concentrations ranging from 0.1 mM to 100 mM K^+^. The K^+^ titration curve was fitted with a one-site saturation model, yielding an apparent dissociation constant (*K*_d_) of 1.66 ± 0.05 mM and an apparent Hill coefficient (*n*_H_) of 1.05 ± 0.03 (Fig. 1d). To test the selectivity for K^+^ relative to Na^+^, we incubated KIRIN1 with Na^+^ at concentrations from 1 µM to 150 mM. The emission ratio remained unchanged upon the addition of Na^+^ even at 150 mM (Fig. 1d), indicating that KIRIN1 has a high degree of selectivity for K^+^ over Na^+^. Furthermore, KIRIN1 exhibited no significant FRET ratio change upon the addition of physiologically relevant concentrations of other ions including Mg^2+^ (10 mM), Ca^2+^ (10 µM), and Zn^2+^ (10 µM) (Fig. 1e), further demonstrating the specificity of KIRIN1 for K^+^. We also carried out kinetic characterization using stopped-flow fluorescence, which revealed a *k*_on_ of 28.5 ± 0.8 mM^−1^ s^−1^ and a *k*_off_ of 105.5 ± 5.3 s^−1^ for K^+^ binding to KIRIN1. These results indicate that KIRIN1 has a rapid kinetic response to changes in K^+^ concentration (Supplementary Fig. 1a).

To compare KIRIN1 with previously published GEPII, we measured the FRET spectral change, K^+^ affinity, and ion specificity of GEPII1.0 in parallel with KIRIN1. GEPII1.0 showed a maximum Δ*R*/*R*_min_ of 220%, which is higher than 130% of KIRIN1. The apparent *K*_d_ for binding K^+^ is similar with GEPII1.0 exhibiting a *K*_d_ of 2.63 ± 0.02 mM and KIRIN1 exhibiting a *K*_d_ of 1.66 ± 0.05 mM. Both indicators had highly similar selectivity toward K^+^ over other physiologically relevant ions as expected (Supplementary Fig. 2).

**Fig. 2:**
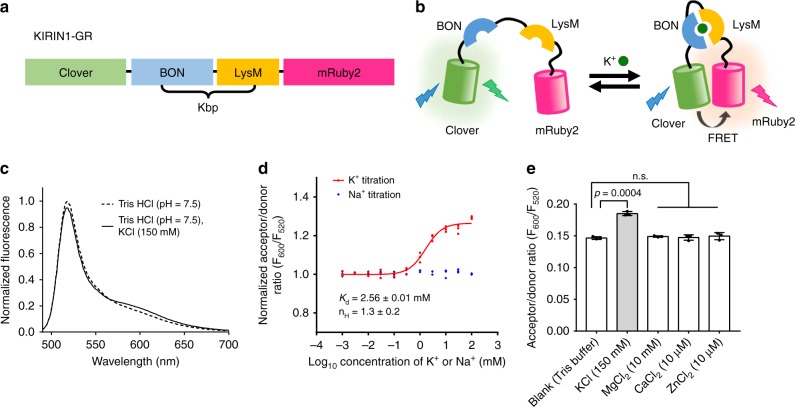
Design and in vitro characterization of KIRIN1-GR. **a** Design and construction of the genetically encoded K^+^ indicator KIRIN1-GR. **b** Schematic of the molecular sensing mechanism of KIRIN1-GR. **c** Emission fluorescence spectrum of KIRIN1-GR with (solid) and without (dashed) K^+^. **d** K^+^ titration curve (red) and Na^+^ titration (blue) of KIRIN1-GR according to FRET acceptor-to-donor fluorescence ratio (*F*_600_/*F*_520_), data are expressed as individual data points. **e** FRET acceptor-to-donor fluorescence ratio of the genetically encoded K^+^ indicator elicited by adding different physiologically relevant ions including Mg^2+^ (10 mM), Ca^2+^ (10 µM), and Zn^2+^ (10 µM), data are expressed as mean ± SD

In a parallel effort, we constructed a red-shifted FRET-based K^+^ indicator using the Clover green fluorescent protein (GFP) and the mRuby2 red fluorescent protein (RFP) pair^38^ (Fig. 2a, b). The resulting indicator, KIRIN1-GR (KIRIN1 with GFP and RFP), exhibited a *K*_d_ of 2.56 ± 0.01 mM for K^+^. Like KIRIN1, it was selective for K^+^, though the in vitro K^+^–induced Δ*R*/*R*_min_ was just 20% (Fig. 2c–e).

### Development of a single fluorescent protein-based K^+^ indicator

**Fig. 3:**
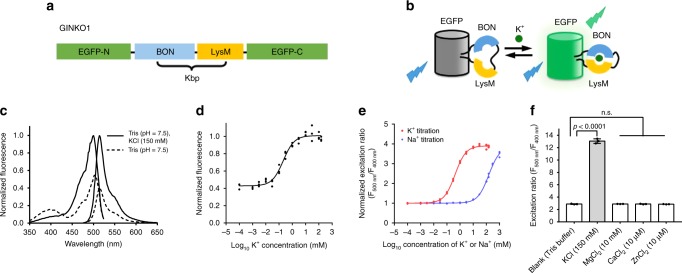
Design and in vitro characterization of GINKO1. **a** Design and construction of the genetically encoded K^+^ indicator GINKO1. **b** Schematic representation of the molecular sensing mechanism. **c** GINKO1 excitation and emission fluorescence spectrum in buffer with (solid) and without (dashed) K^+^. **d** Normalized emission intensity of purified GINKO1 protein at various concentrations of K^+^, data are expressed as individual data points. **e** Excitation ratio of purified GINKO1 protein at various concentrations of K^+^ (red) and Na^+^ (blue) solutions, data are expressed as individual data points. **f** Excitation fluorescence ratio of purified GINKO1 protein upon addition of various physiologically relevant ions, data are expressed as mean ± SD

We next sought to engineer a single fluorescent protein-based K^+^ indicator. We first attempted to engineer such an indicator based on a circularly permutated (cp) enhanced green fluorescent protein (EGFP) scaffold similar to the design of GCaMP^39^ series of Ca^2+^ indicators. In this design, the BON and LysM domains of Kbp were split and fused to the N and C termini, respectively, of cpEGFP. The resulting protein exhibited green fluorescence, but this fluorescence did not change upon addition of K^+^, possibly because the K^+^-binding function of Kbp was abolished by the genetic splitting of the two domains. We next explored an alternate indicator topology by inserting an intact Kbp into a fluorescent protein. This approach has been previously used for Ca^2+^ and glutamate indicators^40–42^. We found that insertion of Kbp into the EGFP scaffold of GCaMP6s at residue 144 yielded a functional green fluorescent indicator for K^+^ (Fig. 3a, b). This indicator was designated as GINKO1.

To characterize GINKO1, we first measured the fluorescence excitation and emission spectrum of purified indicator in Tris buffer with and without K^+^ (Fig. 3c). The excitation and emission maxima of apo-GINKO1 are 502 and 514 nm, respectively. In the K^+^-bound state, the excitation maximum is slightly blue-shifted to 500 nm, most likely due to an alteration of chromophore environment. Upon the addition of 150 mM K^+^, the green fluorescence emission (514 nm) intensity increased to ~250% of its initial intensity (Fig. 3c, d). This increased intensity is attributed to an increase in extinction coefficient from 17,600 M^−1^ cm^−1^ to 37,300 M^−1^ cm^−1^. The quantum yield remained essentially unchanged (0.20 in the absence of K^+^ and 0.21 in the presence of K^+^). GINKO1 also exhibited a K^+^-dependent 3.5-fold change in excitation ratio, making it both an intensiometric and excitation ratiometric indicator (Fig. 3d, e). To determine the K^+^ affinity of GINKO1, we measured the green fluorescence excitation ratio (*F*_500_/*F*_400_) over K^+^ concentrations ranging from 1 µM to 150 mM (Fig. 3e). The K^+^ titration curve revealed an apparent *K*_d_ of 0.42 ± 0.03 mM and an apparent Hill coefficient (*n*_H_) of 1.05 ± 0.03 (Fig. 3e). To test the selectivity for K^+^ relative to Na^+^, we titrated Na^+^ over the concentration range from 1 µM to 1 M (higher concentrations of Na^+^ lead to sensor protein precipitation). GINKO1 fluorescence excitation ratio (*F*_500_/*F*_400_) exhibited an ~3-fold change upon addition of 1 M Na^+^ (Fig. 3e). Fitting of the Na^+^ titration curve revealed an apparent *K*_d_ of 153 ± 8 mM and an apparent *n*_H_ of 1.04 ± 0.05 (Fig. 3e). The apparent *K*_d_ for Na^+^ is over 350 times higher than that for K^+^, indicating that GINKO1 is highly selective towards K^+^ over Na^+^. It is possible that the insertion of Kbp into cpEGFP resulted in a structural change in the ion binding site of Kbp, leading to increased affinity for both K^+^ and Na^+^. GINKO1 exhibited no significant excitation ratio change upon the addition of other ions including Mg^2+^ (10 mM), Ca^2+^ (10 µM), or Zn^2+^ (10 µM) (Fig. 3f), confirming its K^+^ specificity. Stopped-flow kinetic characterization of GINKO1 revealed *k*_on_ = 9.32 ± 0.25 mM^−1^ s^−1^ and *k*_off_ = 85.9 ± 1.6 s^−1^ for K^+^ binding, similar to the kinetic constants for KIRIN1 (Supplementary Fig. 1b).

### Imaging intracellular K^+^ in HeLa cells

**Fig. 4:**
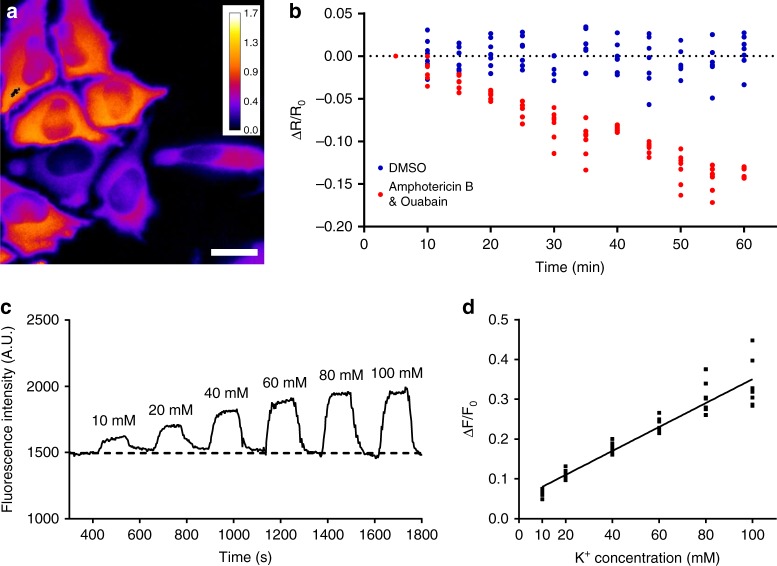
Imaging intracellular K^+^ in HeLa cells. **a** Representative FRET acceptor (excitation 438/24 nm, emission 544/24 nm) to donor (excitation 436/20 nm emission 483/32 nm) fluorescence ratio (*R* = *F*_acceptor_/*F*_donor_) image of live HeLa cells expressing KIRIN1 (scale bar = 20 µm). **b** Trace (red) of FRET acceptor-to-donor fluorescence ratio change (Δ*R*/*R*_0_) after treatment of live HeLa cells expressing KIRIN1 with 5 µM amphotericin B and 10 µM ouabain (*n* = 7), trace (blue) of FRET acceptor-to-donor fluorescence percentage ratio change (Δ*R*/*R*_0_) without chemical treatment (*n* = 13) on live HeLa cells expressing KIRIN1, data are expressed as individual data points. **c** Representative trace for a HeLa cell permeabilized using valinomycin for K^+^ and perfused with a series buffer with different K^+^ concentrations for in situ calibration of GINKO1. **d** Fluorescence intensity changes (Δ*F*/*F*_0_) (*n* = 7) plotted against corresponding known K^+^ concentrations, data are expressed as individual data points

The in vitro characterization suggested that Kbp-based genetically encoded K^+^ indicators could be utilized for visualizing intracellular K^+^ dynamics in live cells. To test the performance of KIRIN1, HeLa cells (an immortalized human cervical cancer cell line) were transfected with the plasmid encoding KIRIN1. Changes in intracellular K^+^ concentrations were then induced pharmacologically using amphotericin B, an antifungal drug that induces K^+^ efflux, and ouabain, an inhibitor of sodium-potassium ion pump (Na/K-ATPase). Addition of both amphotericin B and ouabain to HeLa cells has been reported to deplete intracellular K^+^ content^43,44^. Cells were imaged in both the CFP (excitation 436/20 nm emission 483/32 nm) and FRET (excitation 438/24 nm, emission 544/24 nm) channels, and the FRET ratio image of KIRIN1 was calculated by dividing the FRET channel image by the CFP channel image (Fig. 4a). Upon treatment with 5 µM amphotericin B and 10 µM ouabain, the indicator exhibited a gradual decrease of 15% in FRET ratio over a period of ~60 min, signaling a decrease of K^+^ concentrations in cells (Fig. 4b). The control experiment using KIRIN1-expressing cells treated with dimethyl sulfoxide vehicle revealed no significant change in FRET ratio over the same time period (Fig. 4b). Under similar testing conditions KIRIN1-GR gave an 8% decrease in FRET ratio upon amphotericin B and ouabain treatment (Supplementary Fig. 3). These findings demonstrate that KIRIN1 and KIRIN1-GR are capable of monitoring intracellular K^+^ changes across a population of live cells in real time.

To characterize the capability of GINKO1 for measuring intracellular K^+^, we expressed the gene in HeLa cells via transient transfection. Cells were permeabilized with K^+^-specific ionophore valinomycin, which enables the K^+^ concentrations in cells to be externally manipulated. Solutions of different K^+^ concentrations (10 to 100 mM) were perfused sequentially. In between each perfusion, cells were washed with culture medium containing 3 mM K^+^. The GINKO1 fluorescence intensity was recorded (Fig. 4c) and the fluorescence intensity changes (Δ*F*/*F*_0_) were plotted against the extracellular K^+^ concentrations (Fig. 4d). GINKO1 fluorescence intensity increased in proportion to K^+^ concentrations over the range of 3 mM to 100 mM. This result demonstrates the potential of using GINKO1 to detect and potentially quantify a wide range of K^+^ concentrations in cells.

### Imaging intracellular K^+^ dynamics in neurons with KIRIN1

**Fig. 5:**
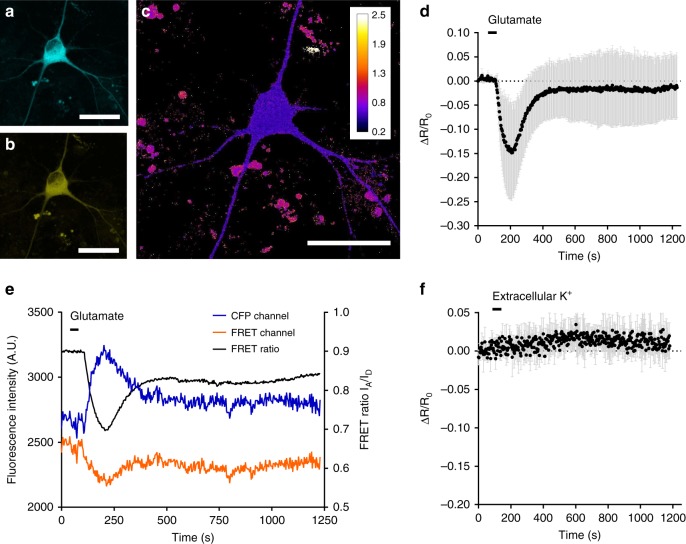
Imaging neuronal K^+^ dynamics using KIRIN1. **a** Representative donor channel image of dissociated cortical neuron expressing KIRIN1 (scale bar = 30 µm). **b** Representative FRET channel image (scale bar = 30 µm). **c** Representative FRET ratio image (scale bar = 30 µm). **d** FRET acceptor-to-donor fluorescence percentage ratio change time course with the treatment of glutamate (500 µM, *n* = 3), data are expressed as mean ± SD. **e** Representative traces of CFP channel intensity (blue), FRET channel intensity (orange), and FRET acceptor-to-donor fluorescence ratio (black) with the treatment of glutamate (500 µM). **f** FRET acceptor-to-donor fluorescence percentage ratio change time course with treatment with a high concentration (30 mM) of extracellular K^+^ (*n* = 3), data are expressed as mean ± SD

K^+^ plays an essential role in maintaining the normal neuronal physiology. Previous studies^45–50^, using electrode-based measurements of extracellular K^+^ concentration, have revealed that overactivation of glutamate receptors using glutamate mediates a strong K^+^ efflux from neurons. The K^+^ efflux is followed by slower influx to restore membrane potential after glutamate is removed. We examined whether these changes in intracellular K^+^ can be detected optically using KIRIN1. We utilized cultured primary cortical neurons isolated from rat and expressed KIRIN1 using transient transfection. KIRIN1 fluorescence was distributed evenly throughout the cytosol (Fig. 5a–c). After perfusion of glutamate for a short period (30 s), neurons expressing KIRIN1 underwent a 15% decrease in FRET ratio within 200 s. Following washing out of glutamate, the FRET ratio of KIRIN1 returned close to the baseline in ~400 s (Fig. 5d, e), suggesting a slow re-equilibration of intracellular K^+^ concentration in neurons. The observed FRET change is consistent with previous measurements using both electrodes^48^ and a synthetic fluorescent K^+^ indicator dye^12^.

To further confirm that the FRET ratio change was due to K^+^ concentration changes, rather than other cellular changes associated with neuronal activities, we performed the same set of experiments but replaced glutamate stimulation with a high extracellular concentration of K^+^ (30 mM)^51^. This condition depolarizes neurons but does not cause a major K^+^ efflux, possibly due to the fact that the K^+^ gradient across the membrane is reduced and thus the driving force for outward K^+^ current is attenuated^46–50^. Under these conditions, neurons expressing KIRIN1 did not undergo any detectable decrease in FRET ratio (Fig. 5f), confirming that KIRIN1 FRET changes specifically reflect K^+^ dynamics during neuronal activity.

### Concurrent imaging of K^+^ and Ca^2+^ dynamics in neurons

**Fig. 6:**
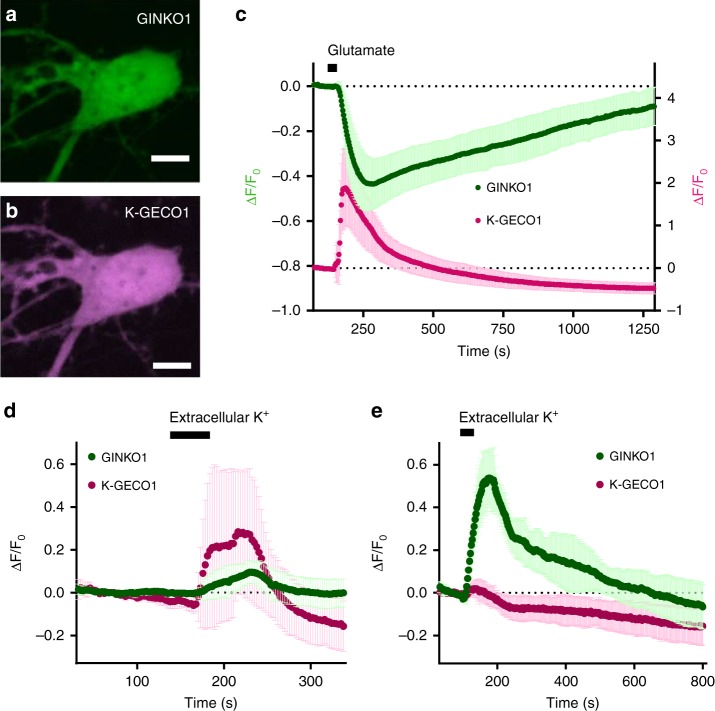
Dual-Color imaging of K^+^ and Ca^2+^ dynamics in cultured cortical dissociated neurons and glial cells. **a** Representative GFP channel fluorescence image of neuron soma region expressing GINKO1 (scale bar = 5 µm). **b** Representative RFP channel fluorescence image of neuron soma region expressing K-GECO1 (scale bar = 5 µm). **c** Normalized fluorescence intensity change (Δ*F*/*F*_0_) time course of GINKO1 and K-GECO1 with stimulation (30 s, 500 µM) of glutamate on neurons (*n* = 10), data are expressed as mean ± SD. **d** Normalized fluorescence intensity change (Δ*F*/*F*_0_) time course of GINKO1 and K-GECO1 with stimulation by a high concentration (30 mM) of extracellular K^+^ on neurons (*n* = 7), data are expressed as mean ± SD. **e** Normalized fluorescence intensity change (Δ*F*/*F*_0_) time course of GINKO1 and K-GECO1 with stimulation by a high concentration (30 mM) of extracellular K^+^ on glial cells (*n* = 4), data are expressed as mean ± SD

One of the advantages of single fluorescent protein-based indicators is that, relative to FRET-based indicators, they can be readily combined with other fluorescent proteins for multiparameter imaging. To explore whether GINKO1 could be used with a red fluorescent Ca^2+^ indicator for imaging of K^+^ and Ca^2+^ dynamics in the same cell, we co-expressed GINKO1 and K-GECO1 (ref. ^52^) in cultured cortical neurons (Fig. 6a, b). Neuronal activity was evoked by a short exposure (30 s) to glutamate (500 µM). Following this treatment, the red fluorescence from K-GECO1 rapidly increased by an average of 188% within 10 s, followed by a gradual decrease back to baseline in 300 s (*n* = 10) (Fig. 6c, magenta trace). The green fluorescence of GINKO1 underwent a relatively slow, but substantial, decrease of 43% in 120 s, and then recovered back to baseline level after 900 s (*n* = 10) (Fig. 6c, green trace), consistent with the results from KIRIN1 in neurons with glutamate stimulation.

We further imaged neuronal K^+^ and Ca^2+^ changes with stimulation using high extracellular concentrations of K^+^ (30 mM). Cultured dissociated cortical neurons were perfused for 60 s with 30 mM extracellular K^+^. Following the stimulation, a 28% increase in K-GECO1 red fluorescence was observed due to neuronal depolarization and Ca^2+^ influx (*n* = 7) (Fig. 6d, magenta trace). In the same cells, GINKO1 green fluorescence exhibited a 9% increase (Fig. 6d, green trace), indicating a K^+^ influx. This K^+^ influx was not detected by KIRIN1 under the same experimental conditions, suggesting that GINKO1 is a more sensitive indicator for reporting intracellular K^+^ concentration elevation.

Building upon this insight, we continued to explore the K^+^ and Ca^2+^ dynamics in (astrocytic) glial cells using GINKO1 and K-GECO1. Compared to neurons, glial cells have relatively hyperpolarized resting membrane potentials and high K^+^ permeability^53,54^. These electrophysiological properties of glial cells contribute to their function in maintaining and regulating extracellular K^+^ concentration. Accordingly, an elevated extracellular K^+^ concentration should trigger K^+^ uptake by glial cells, leading to an increase in intracellular K^+^ concentration^49,50,55,56^. Glial cells expressing both GINKO1 and K-GECO1 were stimulated using 30 mM extracellular K^+^ for 60 s. Upon treatment, GINKO1 green fluorescence intensity increased by 53% (*n* = 4) (Fig. 6e, green trace), a more substantial increase than the 9% increase observed for identically treated neurons. K-GECO1 red fluorescence showed a slight increase of 4% (*n* = 4) (Fig. 6e, magenta trace). These results support the notion that glial cells regulate extracellular K^+^ homeostasis^57^, and also reveal the differential response of glial cells versus neurons in handling K^+^ and Ca^2+^ under depolarized conditions.

## Discussion

We have developed a genetically encoded FRET-based K^+^ indicator, KIRIN1, based on bacterial Kbp and the FRET pair of mCerulean3 and cp173Venus. Characterization of KIRIN1 revealed that the FRET efficiency changes over the physiologically relevant range of K^+^ concentrations. The dynamic range (based on FRET emission ratio) of KIRIN1 in vitro is 130%, which is comparable to some optimized FRET-based indicators^58,59^. Importantly, KIRIN1 is much more selective for K^+^ than Na^+^, due to the inherent K^+^ selectivity of Kbp^15^. Our findings on KIRIN1 are largely consistent with the similarly designed K^+^ indicator, GEPII1.0, which also uses Kbp and a cyan/yellow FRET pair^33^. The main difference between GEPII1.0 and KIRIN1 is the use of mseCFP versus mCerulean3, respectively, as FRET donors. Both indicators use Kbp as a sensing domain and cp173Venus as a FRET acceptor. In addition to KIRIN1, we constructed another K^+^ indicator, KIRIN1-GR, based on the green and red fluorescent protein FRET pair Clover/mRuby2. However, the change in FRET ratio of KIRIN1-GR is substantially smaller than that of KIRIN1. The decreased FRET ratio change may be due to the relatively low quantum yield of the mRuby2 (0.38) FRET acceptor^60^ and the absence of a weak dimerization effect, which is present in the CFP/YFP pair^61,62^. Nonetheless, due to its red-shifted spectrum and large difference in fluorescence hue between donor and acceptor, the GFP/RFP FRET-based KIRIN1-GR may offer a high signal-to-background ratio with minimal spectral bleed-through^60^. Future efforts to increase the KIRIN1-GR FRET change could be directed at replacing the Clover/mRuby2 FRET pair with brighter GFPs such as mClover3 or mNeonGreen, and brighter RFPs such as mRuby3 or mScarlet^38,63,64^. These findings provide further support for the conclusion of Bischof et al.^33^, who found that Kbp-based FRET K^+^ indicators are well suited for sensing K^+^ in vitro and in live cells.

We have also developed a single fluorescent protein-based green fluorescent K^+^ indicator, GINKO1, which was constructed by inserting the Kbp domain into an EGFP scaffold. K^+^ binding induces conformational changes of Kbp and modulation of the fluorescent protein fluorescence, which is most likely associated with alteration of the equilibrium between the protonated and deprotonated forms of the EGFP chromophore^65^. This design enables the detection of K^+^ dynamics based on single emission wavelength fluorescence intensity changes. In vitro characterization of GINKO1 revealed that it has 2.5-fold fluorescent intensity change (*F*_max_/*F*_min_) upon K^+^ binding. Titration revealed that GINKO1 also responds to very high concentrations of Na^+^. Single fluorescent protein-based indicators offer several advantages relative to their FRET-based counterparts. Specifically, they can be more easily used for simultaneous multiparameter imaging with minimal spectral bleed-through. In addition, single fluorescent protein-based indicators have the potential to be engineered for larger intensity-based fluorescence changes, as demonstrated by the highly effective optimization of the GCaMP series of Ca^2+^ indicators^66^. Beyond the typical advantages of single fluorescent protein-based indicators, GINKO1 is also excitation ratiometric. With an approximately 4-fold K^+^ binding-induced excitation ratio change (*R*_max_/*R*_min_), GINKO1 is suitable for ratiometric imaging. Ratiometric imaging is preferable for quantitative imaging as it enables experimental correction for intensity differences that are due to variability in protein expression levels. Coupling GINKO1 with an ion-insensitive fluorescent marker (in a distinct spectral channel) could also correct for such imaging artifacts. For these reasons, we anticipate that further optimized versions of GINKO1 will ultimately be the most versatile tools for K^+^ imaging, and likely far surpass the performance of the FRET-based counterparts, KIRIN1 and GEPIIs.

Single fluorescent protein-based GINKO1, FRET-based KIRIN1, and the previously reported FRET-based GEPIIs^33^ are indicators with large dynamic range and high specificity, making them promising probes for K^+^ imaging. Our imaging experiments in cell lines and dissociated neurons have demonstrated that both KIRIN1 and GINKO1 are useful for imaging K^+^ dynamics intracellularly. The advent of Kbp-based K^+^ biosensors will open new avenues for investigation of cellular signaling associated with normal or abnormal K^+^ dynamics at the single-cell level or across large cell populations. As they are genetically encoded, these indicators are compatible with in vivo expression in animal models, either with viral vectors or through transgenic technology. Potential future applications include imaging K^+^ spatial buffering in glial cells^56^, studying extracellular K^+^ dynamics during cortical spreading depression^67^ and sleep/wake cycle^68^, and imaging K^+^ efflux during macrophage innate immunity NLRP3 activation^69^. Application of K^+^ indicators to these problems, and others, will ultimately provide a deeper understanding of the role of K^+^ dynamics in vivo.

## Methods

### Plasmid DNA construction

DNA encoding KIRIN1 was synthesized and cloned into the pET28a expression vector by GenScripts. For KIRIN1-GR, the Kbp sequence was synthesized by Integrated DNA Technologies (IDT) as a double-stranded DNA fragment; DNA encoding Clover and mRuby2 was obtained from Addgene (plasmid #49089). For GINKO1, the DNA sequence was synthesized by IDT as a double-stranded DNA fragment. For GEPII1.0, plasmid was purchased from NGFI Next Generation Fluorescence Imaging GmbH. DNA fragments of GINKO1 and GEPII1.0 were PCR amplified using Phusion polymerase and then cloned into the pBAD/His-B expression vector (Thermo Fisher Scientific) using Gibson assembly (New England Biolabs). KIRIN1, KIRIN1-GR, and GINKO1 genes were PCR amplified, digested, and ligated into the mammalian expression vector pcDNA3.1 (Thermo Fisher Scientific). pcDNA3.1-NES-KIRIN1 was constructed by PCR with the primer containing the nuclear exclusion signal (NES) encoding the sequence (LALKLAGLDIGS). All constructs were verified by DNA sequencing.

### In vitro characterization

The indicator proteins were expressed as His-tagged recombinant proteins in *E*. *coli*, with an induction temperature of 30 °C overnight. Bacteria were harvested at 10,000 rpm, 4 °C for 10 min. Cell pallet was lysed using sonication, and then clarified using centrifuge at 14,000 rpm for 30 min. The protein was purified from the supernatant by affinity chromatography using Ni-NTA agarose resin (McLab). Eluted protein solution was then buffer exchanged using a PD-10 (GE Healthcare Life Sciences) desalting column. For K^+^ *K*_d_ determination, purified protein was diluted into a series of buffers with K^+^ concentration ranging from 0 to 150 mM. The fluorescence spectrum of the KIRIN1 in each solution (100 µL) was measured using a Tecan Safire2 microplate reader with excitation at 410 nm and emission from 430 nm to 650 nm. The fluorescence spectrum of the KIRIN1-GR in each solution (100 µL) was measured using a Biotek microplate reader with excitation at 470 nm and emission from 490 nm to 700 nm. The fluorescence spectrum of the GINKO1 in each solution (100 µL) was measured using a Tecan Safire2 microplate reader. Titration experiments were performed in triplicate. The FRET ratios for KIRIN1 and KIRIN1-GR or excitation ratios for GINKO1 were plotted as a function of K^+^ concentrations. Graphpad Prism software was used to fit the data and obtain apparent *K*_d_ and the apparent Hill coefficient for K^+^ binding. The brightness of GINKO1 was measured in the presence and absence of K^+^. The purified GINKO1 protein was subject to buffer exchange to either K^+^ and Na^+^ free 50 mM Tris buffer or 150 mM KCl, 50 mM Tris buffer with PD-10 columns (GE Healthcare Life Sciences). The concentration of GINKO1 was determined with base denaturation^36^ and the absorbance was measured with Backman Coulter DU 800 spectrophotometer to determine the extinction coefficient. The quantum yield was measured with EGFP as the control and the fluorescence was measured with a Tecan Safire2 microplate reader. Data were processed with Microsoft Excel. Stopped-flow kinetic measurements were performed on a SX20 Stopped-Flow Reaction Analyzer (Applied Photophysics). For KIRIN1, the excitation wavelength was 430 nm with 2 nm bandwidth and collected emitted light at 530 nm through a 10 mm path. For GINKO1, the excitation wavelength was 488 nm with 2 nm bandwidth and collected emitted light at 520 nm through a 10 mm path. A total of 200 data points were collected over 5 replicates throughout the reaction and the fluorescence changes were examined for 250 ms. Reactions were initiated by mixing equal volumes of diluted purified protein in Tris buffer (100 mM, pH = 7.50) and varying concentrations of KCl (2, 10, and 20 mM) at 20 °C. Data were analyzed with Graphpad Prism to obtain the observed rate constant *K*_obs_ and determine rate constants of association *k*_on_ and dissociation *k*_off_.

### Live cell imaging

For K^+^ imaging of mammalian cell line, HeLa cells were maintained in Dulbecco’s modified Eagle's medium with 10% fetal bovine serum (FBS, Thermo Fisher Scientific), penicillin–streptomycin antibiotics (Thermo Fisher Scientific), and GlutaMAX (Thermo Fisher Scientific) at 37 °C with 5% CO_2_. Hela cells with 60–70% confluence on 35 mm glass-bottom dishes (In Vitro Scientific) were transfected using Polyjet (SignaGen), Turbofect (Thermo Fisher Scientific), or Lipofectamine 2000 (Thermo Fisher Scientific) according to the manufacturer’s instructions. Cells were imaged 18–24 h after transfection. Medium was replaced with 1 mL of 20 mM HEPES buffered Hanks' balanced salt solution (HBSS; HHBSS) immediately before imaging. Imaging was performed with a Zeiss Axiovert 200 microscope with a 40× objective. HeLa cell images were acquired with Molecular Devices MetaMorph and processed using ImageJ.

For K^+^ imaging of cultured dissociated neurons and (likely astrocytic) glial cells, dissociated E18 Sprague Dawley cortical neurons were grown on 35 mm glass-bottom dishes containing NbActiv4 medium (BrainBits LLC) with 2% FBS and penicillin–streptomycin antibiotics at 37 °C and 5% CO_2_. Half of the culture medium was replaced with fresh medium every 4–5 days. Neurons were transfected on day 8 using Lipofectamine 2000 according to the manufacturer’s instructions, with the following modifications. Briefly, 1–2 μg of plasmid DNA and 4 μL of Lipofectamine 2000 were added to 100 μL of NbActive4 medium to make the transfection medium. Half of the culture medium was removed and combined with an equal volume of fresh NbActiv4 medium to make a 1:1 mixture. All dishes were replenished with conditioned (at 37 °C and 5% CO_2_) NbActiv4 medium. Following the addition of transfection medium, cells were incubated at 37 °C and 5% CO_2_ for 2–3 h. The medium was then replaced using conditioned 1:1 mixture medium. Cells were imaged 36–72 h after transfection. Fluorescence imaging was performed in HHBSS buffer using a Zeiss microscope with a 40× objective or Olympus FV1000 microscope with a 20× objective. Neuron images were acquired and processed using Olympus FluoView.

### Statistical analysis

All data are expressed as individual data points or mean ± SD. Sample sizes (*n*) are listed for each experiment. For Figs. 1e, 2e, 3f, and Supplementary Fig. 1c, *t*-tests were used with *p* values indicated in the figures.

## Supplementary information

Description of Additional Supplementary Files

Supplementary Information

Supplementary Movie 1

Supplementary Movie 2

Supplementary Data 1

## Data Availability

The data supporting this research are available in Supplementary Data 1. Plasmid constructs encoding KIRIN1, KIRIN1-GR, and GINKO1 are available through Addgene.
