# Supplementary material for: Genetically encoded fluorescent indicators for imaging intracellular potassium ion concentration
Source: Commun Biol. 2019 Jan 14;2:18. doi: 10.1038/s42003-018-0269-2 (PMC6331434; doi:10.1038/s42003-018-0269-2)
Supplement: Supplementary file 1 — Description of Additional Supplementary Files [file 42003_2018_269_MOESM1_ESM.docx]

**Description of Additional Supplementary Files**

**File Name**: Supplementary Data 1

**Description**: All source data underlying the graphs and charts presented in the main figures are listed in Excel format.

**File Name**: Supplementary Movie 1

**Description**: Imaging intracellular K+ depletion using KIRIN1

**File Name**: Supplementary Movie 2

**Description**: Dual-Color imaging of K+ and Ca2+ dynamics in cultured cortical dissociated neuron.
