# Supplementary material for: Genetically encoded fluorescent indicators for imaging intracellular potassium ion concentration
Source: Commun Biol. 2019 Jan 14;2:18. doi: 10.1038/s42003-018-0269-2 (PMC6331434; doi:10.1038/s42003-018-0269-2)

Supplementary figure 1. Stopped-flow kinetic characterization of genetically encoded K^+ indicators. Observed rate constants (k_{obs}) are plotted as a function of K^+ concentration for (**a**) KIRIN1 and (**b**) GINKO1. Rate constants of association and dissociation (k_{on} and k_{off}) were determined by fitting linear regression to the equation $k_{obs} = k_{on}[K^+] + k_{off}$.

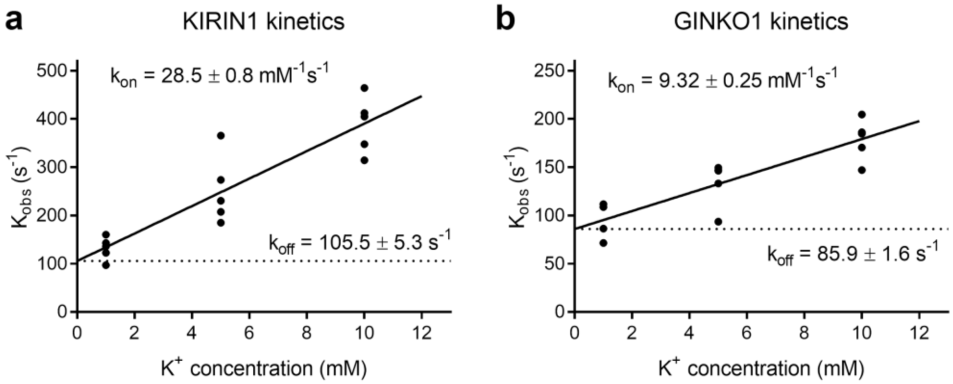

Supplementary figure 2. *in vitro* characterization of GEPII1.0. (**a**) Emission fluorescence spectrum of GEPII1.0 with (red) and without (blue) K^+. (**b**) K^+ titration curve (red) and Na^+ titration (blue) of GEPII1.0 according to FRET acceptor-to-donor fluorescence ratio (F_{530}/F_{475}), data are expressed as mean ± SD. (**c**) FRET acceptor-to-donor fluorescence ratio of the genetically encoded K^+ indicator elicited by adding different physiologically relevant ions including Mg^{2+} (10 mM), Ca^{2+} (10 µM), and Zn^{2+} (10 µM), data are expressed as mean ± SD.

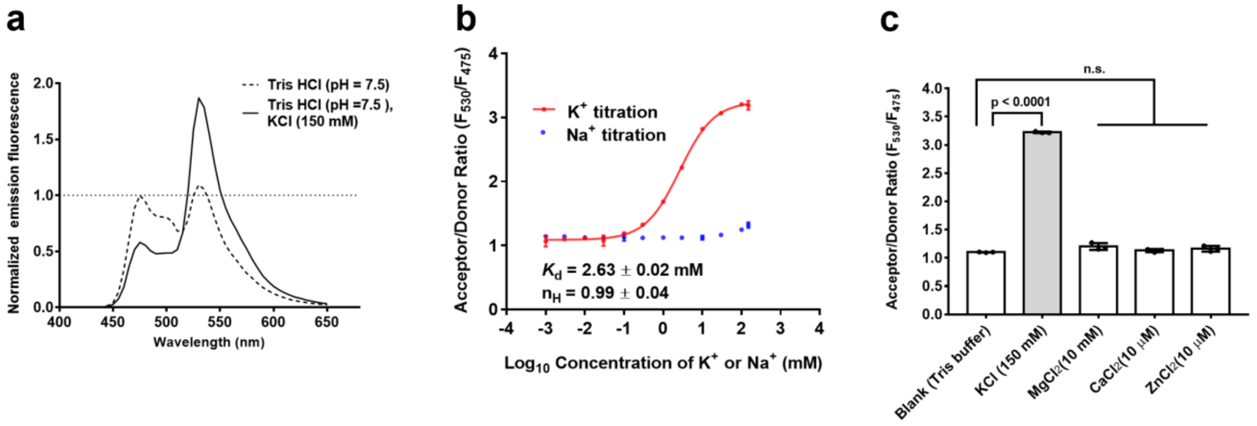

Supplementary figure 3. Imaging intracellular K^+ depletion using KIRIN1-GR. (**a**) Representative FRET acceptor to donor fluorescence ratio (R = $F_{acceptor}/F_{donor}$) image of live HeLa cells expressing KIRIN1 (scale bar = 20 µm). (**b**) Trace (red) of FRET acceptor-to-donor fluorescence ratio change ($\Delta R/R_0$) after treatment of live HeLa cells expressing KIRIN1-GR with 5 µM amphotericin B and 10 µM ouabain (n = 5), trace (blue) of FRET acceptor-to-donor fluorescence percentage ratio change ($\Delta R/R_0$) without chemical treatment (n = 5) on live HeLa cells expressing KIRIN1-GR, data are expressed as mean ± SD.

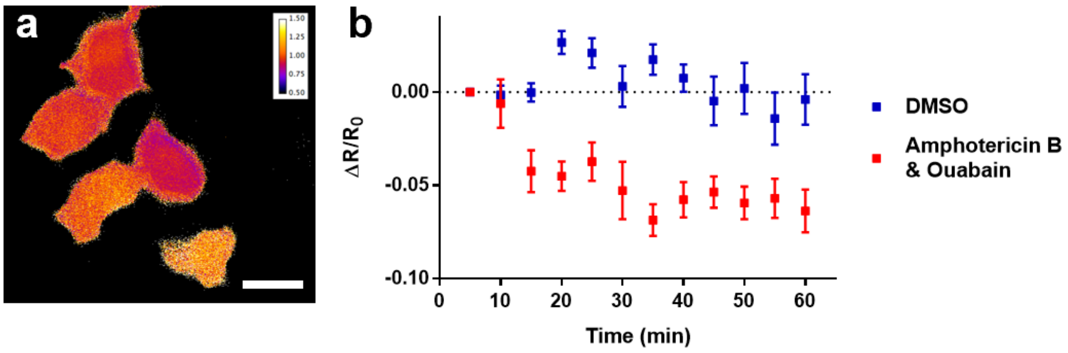

Supplement: Supplementary file 2 — Supplementary Information [file 42003_2018_269_MOESM2_ESM.pdf]
